# Ionic Liquids as Environmental Pollutants—Analysis of the Effect of Tetrabutylammonium Chloride on the Growth and Development of Wheat and Cucumber

**DOI:** 10.3390/toxics11060522

**Published:** 2023-06-09

**Authors:** Barbara Pawłowska, Dagmara Wojtala, Robert Biczak

**Affiliations:** The Faculty of Science and Technology, Jan Dlugosz University in Czestochowa, 13/15 Armii Krajowej Av., 42-200 Częstochowa, Poland; dagmara.wojtala16@gmail.com (D.W.); r.biczak@ujd.edu.pl (R.B.)

**Keywords:** phytotoxicity, chlorophyll fluorescence, photosynthetic pigments content, ionic liquids

## Abstract

Ionic liquids are a huge group of chemical compounds that have found, or may, in the future, find, applications in various industries. These compounds are characterized by excellent physical, chemical, and biological properties, but a big problem is their environmental impact. One of the representatives of this group of compounds is tetrabutylammonium chloride ([TBA][Cl]). In this present study, the effects of [TBA][Cl] were evaluated on two popular plant species—a monocotyledonous plant—wheat (*Triticum aestivum* L.) and a dicotyledonous plant—cucumber (*Cucumis sativus* L.). The results showed that the compound caused a pronounced inhibition of plant growth and roots, as well as plant fresh weight yield. An increase in plant dry weight was observed at the same time. Despite the decrease in the content of photosynthetic pigments, no major changes were observed in chlorophyll fluorescence. All observed changes were strongly related to the applied concentration of the compound.

## 1. Introduction

Ionic liquids (ILs) are compounds that have been of tremendous interest to researchers around the world for more than two decades. A major advantage is their non-volatility, making them safe for the atmosphere and an interesting alternative to volatile organic compounds. Thanks to the enormous possibility of cation–anion combinations, these compounds are often referred to as “designer solvents”. This allows cations and anions to be selected in such a way as to result in compounds with the desired physical, chemical, and biological properties. They can be used in liquid–liquid separations, extraction, fuel cells, pharmaceuticals, lubricants, and in the manufacture of materials such as gels and membranes. However, despite their excellent properties and safety for the atmosphere, it very quickly became apparent that these compounds can exhibit harmful effects on various environmental elements. Numerous studies have shown that ILs, depending on their structure, sometimes exhibit very strong bactericidal, fungicidal, and herbicidal effects. When released into the environment, these compounds can act as toxic substances to, among other things, crops and various types of water- or soil-dwelling organisms. In addition, they can accumulate in the environment, as they are often characterized by high persistence and poor biodegradability [1,2,3,4,5,6,7,8,9].

The use of ILs in the industry may cause these compounds to enter the environment with wastewater, among other things. Additionally, the use of these compounds in pharmaceuticals can cause them to enter the environment along with medicines. In addition to contaminating water and sewage sludge, these compounds can end up in the soil. ILs can be retained in the soil via soil sorption. They can also be taken up by plants. If a plant absorbs ILs, it can affect its growth and development and, consequently, the size and quality of the crop. In addition, it should be remembered that most cultivated plants are used as food for humans and animals. The uptake of harmful compounds by such plants can, therefore, pose a serious threat to consumers [7,10,11]. Despite the vast amount of research available at the moment, the behavior of the cation and anion that enter the environment is still unknown, and the mechanism of how ILs affect plants is not fully known [12].

Therefore, to evaluate the impact of ILs on crops, two popular crop species were selected: a monocotyledonous plant, wheat, and a dicotyledonous plant, cucumber. Wheat is one of the staple crops grown around the world. In addition to the grain, which provides food for both humans and animals, bran and straw are also used. Cucumber, on the other hand, is a plant from the cucurbit family, which is one of the dicotyledonous plants grown worldwide for food. The vegetable is also used in cosmetics.

Tetrabutylammonium chloride [TBA][Cl] was chosen as a typical representative of ionic liquids. This compound is used in chemical synthesis as a catalyst, among other things. At the moment, there are no papers in the scientific literature on the effects of [TBA][Cl] on wheat and cucumber. However, there are reports related to the effects of individual ions included in this compound. As reported by Zhu et al. [13,14], among others, chloride ions have a significant effect on the growth and development of cucumber seedlings. However, we know from numerous articles that the ions combined into an ionic liquid do not behave as is the case with ordinary inorganic salts. Liu et al. [15] studied the growth inhibition of the green alga *Scenedesmus obliquus* by five ionic liquids of 1-alkyl-3-methylimidazolium chloride ([Cnmim]Cl, *n* = 6, 8, 10, 12, 16). They ranked the toxicity of the tested compounds to algae as [C6mim]Cl < [C8mim]Cl < [C10mim]Cl < [C12mim]Cl <[C16mim]Cl. This, therefore, shows that the toxic effect of ILs is influenced by the entire compound, not just the anion or cation itself. In the case of the indicated study, it was the length of the alkyl chain in the cation that had a decisive effect on the toxicity of the tested compounds. At the same time, studies available indicate different effects of anions on the toxicity of ILs. Cho et al. [16], studying the effects of selected imidazole ILs on *Selenastrum capricornutum*, ranked the toxicity of anions according to the following scheme: SbF_6_^−^ > PF_6_^−^ > BF_4_^−^ > CF_3_SO_3_^−^ > C_8_H_17_OSO_3_^−^ > Br^−^ = Cl^−^. In view of the above, further research seems necessary to determine the effects of specific compounds on different plant species.

The presented research was conducted under laboratory conditions in soil culture. Very often, hydroponic cultivation is used for this type of research. However, this type of cultivation does not consider the actual cultivation of plants in the soil, as it ignores any interactions in the soil [5]. From previous studies, it can be observed that the results on the effects of ILs on plants grown under hydroponic conditions and in soil culture differ. Therefore, despite the greater difficulty in conducting this type of research and interpreting the results obtained, the authors investigated the effect of the presence of [TBA][Cl] in the soil on wheat and cucumber.

## 2. Materials and Methods

### 2.1. Materials

Tetrabutylammonium chloride ([TBA][Cl]) (purity ≥ 97%) was purchased from Sigma-Aldrich Chemical Co. This compound occurs as a solid with a melting point of 47–50 °C. It is well soluble in water.

### 2.2. Conditions for Conducting the Pot Experiment

The experiments presented in this paper were carried out in a vegetation hall in accordance with the guidelines contained in the OECD/OCDE 208/2006 guide [17] and the PN-EN ISO 11269–2 standard [18]. In the pot experiment, the effect of [TBA][Cl] on two plant species wheat (*Triticum aestivum* L.) cv. Dawn and cucumber (*Cucumis sativus* L.) cv. Polan were determined. Twenty wheat seeds and ten cucumber seeds each were sown into pots filled with control soil (without ILs) or soil with [TBA][Cl] at concentrations of 1, 10, 100, 400, 700, and 1000 mg∙kg^−1^ of soil DW (dry weight). The soil used in the experiment was loamy sand. Organic carbon content was 8.5 g kg^−1^, soil pH (KCl) = 6.7. The research was conducted under constant, controlled conditions. The temperature during the conduct of this study was 20 ± 2 °C. The substrate moisture content was also kept constant at 70% ppw. Illumination was carried out under a 16 h day/8 h night regime at a level of 170 μmol·m^−2^·s^−1^. All tests were carried out in four replicates. Fourteen days after sowing the seeds into the soil, photographs were taken of the plants under study, and the material, on which the various analyses were performed, was collected.

To assess the effect of [TBA][Cl] on plants, the evaluation of plant fresh weight yield, the inhibition of the length of aboveground parts of plants and roots, dry matter content, and chlorophyll content and its fluorescence were used. Changes in the appearance of plants and their roots were also observed, which were documented in digital photos.

Inhibition of the length of the above-ground parts of the plants and their roots was determined according to the methodology described by Wang et al. [19]. The inhibition factor was calculated as (length/weight in the control group—length/weight in the group in contact with [TBA][Cl]/length/weight in the control group × 100%. The results were expressed as % inhibition of the length of the aboveground parts of cucumber or wheat plants and the length of the roots of the test plants compared to the control.

To determine the dry matter content, approximately 1 g of fresh plant weight was weighed and dried at 105 °C until a constant weight is obtained [20].

Effective concentrations (EC_50_) were also determined. Calculations were performed using nonlinear regression analysis using GraphPad Prism software (GraphPad Software, Inc., La Jolla, CA, USA).

### 2.3. Determination of Photosynthetic Pigments

A total of 0.2 g of fresh leaf mass was homogenized with 80% acetone solution cooled to 4 °C and centrifuged at 15,000 rpm for 10 min. Using a UV-VIS spectrophotometer (V-730 spectrophotometers by Jasco, Krakow, Poland), absorbance at 470 nm, 647 nm, and 664 nm was measured. Photosynthetic pigment content was calculated using appropriate formulas [21].

### 2.4. Chlorophyll Fluorescence

A chlorophyll fluorescence meter type OS1p (GEOMOR TECHNIK, Poland) was used to measure chlorophyll fluorescence. The following were measured: initial (minimum) fluorescence (F_0_), maximum fluorescence (F_m_), and photosynthetic system II (PSII) parameters such as variable fluorescence (F_v_), maximum quantum yield of PSII photochemistry (F_v_/F_m_) and its more sensitive form F_v_/F_0_. For this purpose, blackout clips were placed on the leaves of the plants, which blocked the light supply to these plant parts completely for 30 min. After adaptation in the dark, the sample is irradiated with high-intensity actinic light produced in front of an LED embedded in the fluorimeter. At the same time, the photodetector records the fluorescence of chlorophyll.

### 2.5. Statistical Analysis

STATISTICA 13.3 was used to analyze the results. To determine homogeneous groups, data from four measurements (*n* = 4) were analyzed using one-way ANOVA followed by Tukey’s post hoc test. Significant differences were determined at *p* < 0.05. Results were expressed as arithmetic mean ± standard deviation.

## 3. Results and Discussion

### 3.1. Effect of [TBA][Cl] on Plant Growth and Development

The ionic liquid tested had a significant effect on the growth and development of wheat and cucumber. The plant more sensitive to the presence of [TBA][Cl] in the soil was cucumber. Even the application of a concentration of 10 mg∙kg^−1^ of soil DW caused about a 25% decrease in plant fresh weight yield compared to the control. In the case of wheat, lower concentrations did not cause any visible changes. Only the presence of [TBA][Cl] in the soil at a concentration of 400 mg∙kg^−1^ of soil DW caused about a 37% decrease in yield relative to the control (Table 1).

**Figure 1:**
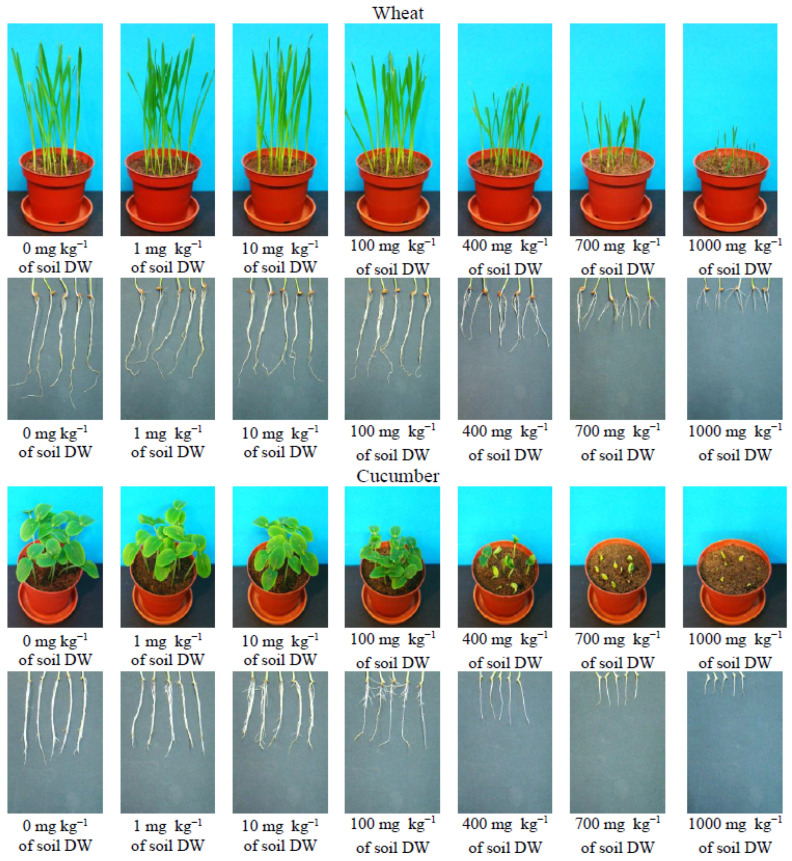
Digital photographs of cucumber and wheat seedlings and roots after introduction to the soil [TBA][Cl].

Other than growth inhibition for wheat, no other changes in plant appearance were observed. For cucumber, when applying concentrations of 700 and 1000 mg∙kg^−1^ of soil DW, the leaves had difficulty releasing from the seed husk or were still partially covered by soil resulting in large chlorotic changes in the leaves (Figure 1).

**Figure 2:**
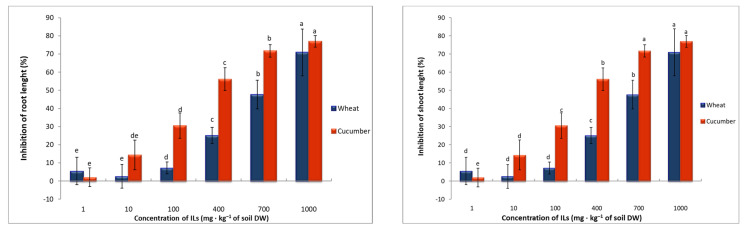
The inhibition rate (%) of root length and shoot length of seedlings of cucumber and wheat after exposure to [TBA][Cl]. Values denoted by the same letters for same biomarkers do not differ statistically at *p* < 0.05.

The reduction in plant fresh weight yield is reflected in the inhibition of the length of the above-ground parts of plants and their roots (Figure 1 and Figure 2). Inhibition of plant aboveground parts was observed in plants growing on soil with ILs at a concentration of 10 mg∙kg^−1^ of soil DW for cucumber and 400 mg∙kg^−1^ of soil DW for wheat. Inhibition of wheat root length was observed when Ils were applied at a concentration of 100 mg∙kg^−1^ of soil DW, as was inhibition of cucumber root length. At the same time, a reduction in the number of lateral roots in cucumbers was observed. At concentrations of 400 mg∙kg^−1^ of soil DW and higher for cucumber, no lateral roots were observed at all (Figure 2).

Based on the inhibition of plant shoots and roots and fresh weight yield, EC_50_ values were determined (Table 2). From the calculated values, it is indisputable that the organ most sensitive to the presence of [TBA][Cl] in the soil was the root.

An important parameter regarding the effect of ILs on plants is changes in dry matter content. In the studies conducted, the presence of [TBA][Cl] in the soil caused an increase in dry matter content. For wheat, these changes were observed after the application of the same concentrations, which caused inhibition of the growth of the above-ground parts of the plants and a decrease in fresh matter yield. For cucumber, an increase in dry matter content was observed, similar to root length inhibition, at a concentration of 100 mg∙kg^−1^ of soil DW. However, the increase in dry matter content was significantly greater for cucumber than for wheat (Table 1).

In contrast, no statistically significant effect of the presence of [TBA][Cl] on seed germination was observed (Table 1).

As shown in previous own studies [22,23], not all ILs show an effect on seed germination. The direction of the effect of ILs on germination depends largely on the type of ILs. However, despite the lack of effect of ionic liquids on seed germination, the compound was taken up from the soil by the plant, resulting in inhibition of the length of the above-ground parts of the plant and its roots, as well as fresh weight yield. Habibul et al. [24] in their study showed that imidazole-based ILs accumulated in roots, while a small amount accumulated in stems and leaves. Trends in the accumulation of ionic liquids by plants depended on the concentration of compound used and the length of the substituent. The accumulation of ILs in individual organs of rice seedlings decreases with increasing alkyl chain length. Chu et al. [5], studying the effects of imidazole ILs on *Isatis tinctoria*, indicate that these compounds applied at low concentrations show no effect on seed germination. The authors showed that ILs up to a certain concentration could stimulate seeds to protect themselves from the negative effects of other compounds present in the soil and accelerate germination. However, a decrease in seed germination was observed after applying higher concentrations of ILs. In addition, as reported by Cvjetko Bubalo et al. [25], ILs containing anions such as BF_4_^−^ and Cl^−^ can be hazardous to the environment due to the possibility of hydrolysis of these compounds occurring, resulting in the release of HCl and HF. These acids can affect germination rates by altering soil pH.

In the discussed studies, the dose–response relationship is very evident, both in the observed inhibition of the length of the above-ground parts of plants and roots, as well as in the yield of plant fresh weight. This observation is confirmed by numerous reports in the literature [19,22,25,26]. Once the seeds have germinated, the organ in direct contact with the soil, and thus with any substances in it, is the root. The presence of [TBA][Cl] in the soil, as previously indicated, caused root length inhibition in cucumber and wheat. In addition, changes in the appearance of the roots were also observed, manifested primarily in the reduction or even complete absence of lateral roots. Chapman et al. [27] believe that the presence of contaminants in the soil can lead to damage to the cell membrane of the roots, which will cause the toxins present in the soil to penetrate unhindered into the roots and through them further into other organs of the plant. The impairment of the root system directly affects the development of the entire plant, as the root is responsible for the uptake of water and nutrients from the soil. As with germination, there is a view among many researchers that low concentrations of ILs may even stimulate faster plant growth, but higher concentrations will cause plants to inhibit root and plant length and yield [10,28,29].

Biomarkers directly related to plant condition also included dry matter content. High concentrations of [TBA][Cl], which affected the development of the root, caused disturbances in the uptake of water and mineral nutrients with the apparent effect of increasing dry matter content in cucumber plants and wheat seedlings. In addition, the introduction of ionic liquids in the form of chlorides into the soil can cause salinization of the soil, which can further disrupt plant water management and result in an increase in plant dry matter [30,31].

### 3.2. Effect of [TBA][Cl] on Photosynthetic Pigment Content

**Figure 3:**
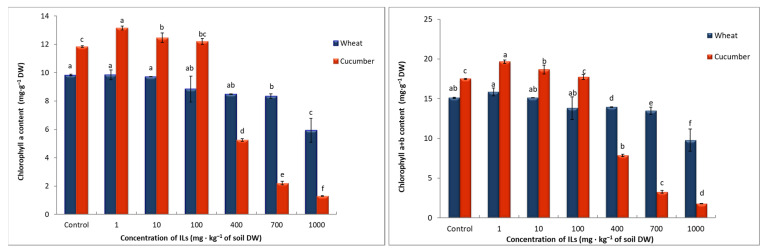

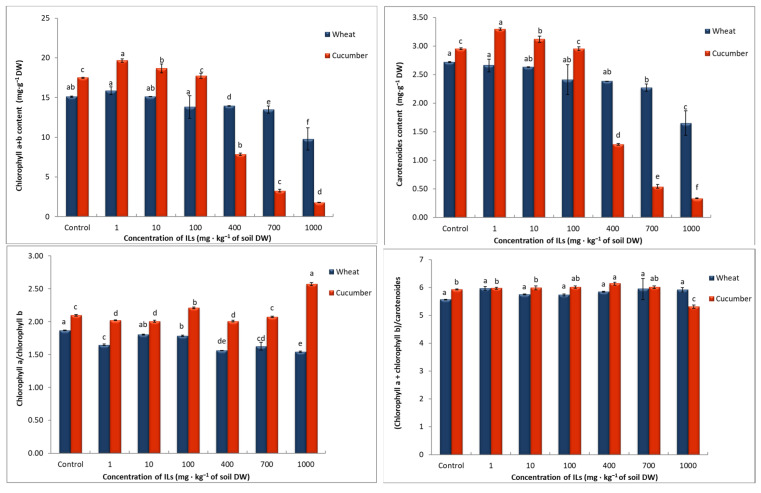
Effect of [TBA][Cl] on the photosynthetic pigment in seedlings of wheat and cucumber. Values denoted by the same letters do not differ statistically at *p* < 0.05.

The amount of yield obtained is inextricably linked to the process of photosynthesis, which provides the plant with the nutrients necessary for proper growth and function. Photosynthetic pigments, which include chlorophylls and carotenoids, play an important role in the process of photosynthesis. In our study, we observed a decrease in the content of chlorophyll a and b, total chlorophyll, and carotenoids in cucumber plants and wheat seedlings growing on soil supplemented with [TBA][Cl]. As with the previously discussed parameters, these changes depended on the applied concentration of the compound (Figure 3).

It was observed that with the application of lower concentrations of compounds, the chlorophyll content in cucumber initially increased compared to the control, but after the application of a concentration of 400 mg∙kg^−1^ of soil DW, the chlorophyll content significantly decreased as the concentration of the compound increased. In wheat seedlings, changes in chlorophyll content were much smaller than for cucumber plants. However, a decrease in the content of photosynthetic pigments positively correlated with the applied concentration of the compound was also observed for this plant. However, no major changes were observed in the ratio of total chlorophyll to carotenoids and chlorophyll a to chlorophyll b. Only the application of a concentration of 1000 mg∙kg^−1^ of soil DW caused a slight increase in the ratio of chlorophyll a to chlorophyll b in cucumber leaves.

The effect of the tested compounds on photosynthetic pigment content is explained by two factors. First, ILs can affect the degradation of chlorophyll molecules. The second reason resulting in a decrease in the content of these pigments in plants is the inhibition of chlorophyll synthesis. The negative effect of ILs on the content of photosynthetic pigments in plants has also been argued by other researchers in their work [19,32,33,34].

As already mentioned in the conducted studies, practically no significant changes were observed in the ratio of total chlorophyll to carotenoids and chlorophyll a to chlorophyll b. Changes in the values of these parameters may indicate the occurrence of oxidative stress in plants. The absence of changes in these ratios observed in this present study does not necessarily indicate the absence of oxidative stress in the experimental plants, as it can only be due to the fact that a percentage of similar decrease in the content of all photosynthetic pigments was observed.

### 3.3. Effect of [TBA][Cl] on Chlorophyll Fluorescence

Photosynthetic pigments that are involved in the process of photosynthesis absorb the energy from the sun, which is necessary to trigger photochemical reactions. The energy that is captured by these pigments is not used in its entirety. Some of the energy absorbed by the pigments is lost as heat or is emitted as chlorophyll fluorescence. Although the emission of fluorescence comes directly from chloroplasts, which contain chlorophyll, it is linked to other metabolic and physiological processes taking place in the plant cell. This means that any changes in the environment in which the plant lives will affect the process of photosynthesis [35]. Very often, even before there are visible symptoms of stress in plants, the effect of stressors on chlorophyll fluorescence can be observed [36].

In order to determine the changes in chlorophyll fluorescence in cucumber and wheat growing on soil supplemented with [TBA][Cl], the following fluorescence parameters were measured: F_0_, F_m_, F_v_, F_v_/F_0_, F_v_/F_m_. The value of F_0_ is the initial (zero) fluorescence, i.e., it is such a level of fluorescence at which it is assumed that all antenna pigment complexes associated with the photosystem are open (adapted to darkness). F_m_ is the maximum fluorescence value, and F_v_ is the fluorescence variable and represents the difference between the maximum and initial fluorescence. F_v_/_Fm_ and F_v_/F_0_ ratios are important parameters of PSII activity [37]. F_v_/F_m_ reflects the maximum quantum yield of the PSII photosystem, while F_v_/F_0_ reflects the potential yield of PSII [38]. In our study, the application of lower concentrations of [TBA][Cl] did not cause any changes in the chlorophyll fluorescence parameters tested, both in cucumber and wheat. Only the application of [TBA][Cl] at a concentration of 400 mg∙kg^−1^ of soil DW for wheat and 100 mg∙kg^−1^ of soil DW for cucumber caused a slight decrease in the values of the F_0_, F_v_, and F_m_ parameters. However, it did not lead to changes in the values of F_v_/F_0_ and F_v_/F_m_ (Table 3). Unfortunately, the presence of [TBA][Cl] in the soil at the highest concentrations, equal to 700 and 1000 mg·kg^−1^ of soil DW caused so much inhibition of plant growth that it was not possible to measure chlorophyll fluorescence.

The parameters F_0_ and F_m_ are related to the physiological state of the PSII reaction center. Both an increase and decrease in these parameters can indicate damage to the PSII photosystem. The reason for the increased F_0_ value may be reversible or irreversible inactivation of PSII or damage to the thylakoid membrane, resulting in inhibition of photosynthesis [39]. The decrease in Fm values may be the result of an increase in non-photochemical quenching caused by photosynthetic disruption. PSII photoinactivation can cause oxidative damage, resulting in the loss of PSII reaction centers and increased F_0_ [40]. The absence of statistically significant changes in F_v_/F_m_ and F_v_/F_0_ values may indicate that the self-defense mechanism against oxidative stress in plant cells is effective and attempts to counteract the stress. Stress factors to which plants are exposed can cause overproduction of reactive oxygen species (ROS). When increased amounts of ROS appear in cells, the antioxidant system is activated. The cellular antioxidant system is a multi-component enzymatic and small-molecule system responsible for the removal of ROS. It consists of a number of enzymes responsible for breaking down ROS, i.e., superoxide dismutase, catalase, or peroxidase operating in the so-called Haliwell-Asada cycle, and low-molecular compounds that readily react with free radicals, i.e., glutathione, carotenoids, ascorbate, ubiquinol or tocopherols. A properly functioning antioxidant system protects the plant from the negative effects of oxidative stress and thus allows the plant to “tolerate” factors harmful to it [22,31]. The results obtained in the presented study were confirmed by the available literature. Liu et al. [34,39,40], Li et al. [32], and Chu et al. [5,6], while studying the effects of ionic liquids on plants of rice, *Arabidopsis thaliana*, *S. obliquus* alga, corn, *Isatis tinctoria*, and *Nostoc punctiforme* alga, also observed changes in individual chlorophyll fluorescence parameters. However, the observed changes did not have a common direction. Depending on the type of ionic liquid, the species of the plant, and its age, an increase or decrease in individual chlorophyll fluorescence parameters was observed. However, all researchers agree that any changes appearing in chlorophyll fluorescence are indicative of stress in plants.

## 4. Conclusions

The results obtained indicate that ionic liquids, of which tetrabutylammonium chloride is representative, can be treated as an environmental pollutant, showing negative effects on plants, i.e., cucumber and wheat. The negative effect on plants depended on the concentration of the compound used. [TBA][Cl] caused inhibition of shoot length, root length, and fresh weight yield of both plants. At the same time, an increase in dry matter content was observed in the tested plants. The tested ionic liquid also showed negative effects on photosynthetic pigment content and photosynthesis in both wheat and cucumber leaves. Cucumber proved to be the plant more sensitive to the ILs tested. These results indicate that ILs can cause oxidative stress in plants and lead to a situation in which plants produce self-defense mechanisms at low concentrations of ILs, while at high concentrations of these compounds in the soil, the cellular structure is destroyed, which leads to disorders in plant development, resulting in inhibition of plant growth and a decrease in yield. Therefore, it is necessary to pay very close attention to the nature of the impact of ionic liquids already used in industry or planned for use on the environment to not lead to contamination of water and soils with these compounds. At the same time, since we know from the available literature that ILs can have varying effects on different species of plants or other organisms, it is necessary to continue this type of research on the determination of such impacts in order to reduce or completely eliminate highly toxic compounds.

## Figures and Tables

**Table 1:** Effect of [TBA][Cl] on emergence, fresh matter yield, and dry matter content in cucumber plants and wheat seedlings.

Concentration of [TBA][Cl] (mg kg^−1^ of Soil DW)	Number of Plants in the Pot	Yield of Fresh Weight (g poot^−1^)	Dry Weight (g g^−1^ FW)
Wheat			
0	20 ±1 ^a^	2.323 ±0.264 ^a^	0.0963 ± 0.0006 ^d^
1	19 ± 0 ^a^	2.139 ± 0.126 ^a^	0.0938 ± 0.0016 ^d^
10	20 ± 1 ^a^	2.371 ± 0.077 ^a^	0.0967 ± 0.0018 ^d^
100	19 ± 1 ^a^	2.156 ± 0.015 ^a^	0.0962 ± 0.0009 ^d^
400	20 ± 1 ^a^	1.463 ± 0.041 ^b^	0.1089 ± 0.0009 ^c^
700	20 ± 1 ^a^	0.792 ± 0.241 ^c^	0.1274 ± 0.0020 ^b^
1000	19 ± 1 ^a^	0.228 ± 0.166 ^d^	0.1651 ± 0.0001 ^a^
Cucumber			
0	10 ± 0 ^a^	3.925 ± 0.104 ^a^	0.0724 ± 0.0011 ^e^
1	9 ± 1 ^a^	3.599 ± 0.216 ^a^	0.07127 ±0.0003 ^e^
10	9 ± 0 ^a^	2.960 ± 0.260 ^b^	0.0745 ±0.0008 ^e^
100	10 ± 1 ^a^	2.567 ± 0.315 ^b^	0.0863 ± 0.0005 ^d^
400	9 ± 1 ^a^	1.054 ± 0.114 ^c^	0.1743 ± 0.0070 ^c^
700	8 ± 1 ^a^	0.580 ± 0.131 ^d^	0.2262 ± 0.0003 ^b^
1000	8 ± 2 ^a^	0.477 ± 0.059 ^d^	0.3034 ± 0.0002 ^a^

Data are means ± SD from four independent experiments. Values denoted by the same letters in the columns do not differ statistically at *p* < 0.05.

**Table 2:** The EC_50_ [mg∙kg^−1^ of soil DW] values for cucumber and wheat seedlings following exposure to [TBA][Cl].

	Wheat	Cucumber
Inhibition for Fresh Weight	973.9	2797
Inhibition for Root Length	476.7	384.6
Inhibition for Shoot Length	1887	2089

**Table 3:** Effect of [TBA][Cl] on F_0_, F_v_, F_m_, F_v_/F_m_, and F_v_/F_0_ in cucumber leaves and wheat seedlings. Values denoted by the same letters in the columns do not differ statistically at *p* < 0.05.

Concentration of [TBA][Cl] (mg∙kg^−1^ of soil DW)	F_0_	F_m_	F_v_	F_v_/F_m_	F_v_/F_0_
Wheat					
0	199.67 ± 8.73 ^a^	1002.50 ± 25.11 ^ab^	802.83 ± 22.06 ^ab^	0.800 ± 0.007 ^a^	4.026 ± 0.182 ^a^
1	200.17 ± 5.04 ^a^	1032.17 ± 45.26 ^a^	832.00 ± 42.74 ^a^	0.805 ± 0.007 ^a^	4.156 ± 0.191 ^a^
10	196.83 ± 8.42 ^a^	1009.50 ± 35.37 ^ab^	812.67 ± 28.05 ^ab^	0.805 ± 0.004 ^a^	4.131 ± 0.102 ^a^
100	197.67 ± 5.43 ^a^	1028.50 ± 23.59 ^a^	830.83 ± 19.43 ^a^	0.807 ± 0.003 ^a^	4.204 ± 0.083 ^a^
400	179.17 ± 13.33 ^b^	943.00 ± 77.42 ^b^	763.83 ± 65.26 ^b^	0.809 ± 0.005 ^a^	4.262 ± 0.157 ^a^
700	-	-	-	-	-
1000	-	-	-	-	-
Cucumber					
0	188.67 ± 7.06 ^a^	990.17 ± 18.17 ^a^	801.50 ± 22.64 ^a^	0.809 ± 0.009 ^a^	4.255 ± 0.255 ^a^
1	188.00 ± 9.84 ^a^	997.33 ± 31.02 ^a^	809.33 ± 23.42 ^a^	0.811 ± 0.006 ^a^	4.310 ± 0.160 ^a^
10	183.00 ± 6.39 ^ab^	977.17 ± 13.61 ^a^	794.17 ± 15.09 ^a^	0.812 ± 0.007 ^a^	4.345 ± 0.200 ^a^
100	175.33 ± 5.20 ^b^	930.67 ± 45.34 ^a^	755.33 ± 42.72 ^a^	0.811 ± 0.008 ^a^	4.308 ± 0.216 ^a^
400	159.50 ± 7.06 ^c^	807.50 ± 80.52 ^b^	648.00 ± 75.24 ^b^	0.802 ± 0.0018 ^a^	4.056 ± 0.374 ^a^
700	-	-	-	-	-
1000	-	-	-	-	-

## Data Availability

The dataset used in this current research is available from the corresponding author upon reasonable request.
